# Using Co-Production to Improve the Local MRCPsych Course at East London NHS Foundation Trust

**DOI:** 10.1192/bjo.2026.11164

**Published:** 2026-06-30

**Authors:** Ivy Lim, Rahul Bhattacharya

**Affiliations:** East London NHS Foundation Trust, London, United Kingdom

## Abstract

**Aims:**

The Membership examination of the Royal College of Psychiatrists (MRCPsych) is a postgraduate professional exam that doctors must pass to progress from core to specialist training in psychiatry in the UK. The MRCPsych courses refer collectively to a range of educational programmes, in England usually organised by different NHS Trusts, funded via postgraduate education through deaneries. There was a move to virtual teaching for the MRCPsych theory papers since the Covid-19 pandemic, providing greater accessibility. This came with challenges as trainers and learners struggled with engagement. This study examined an MRCPsych teaching programme delivered across three trusts, enrolling 121 resident doctors in 2023–2024 and 148 in 2024–2025, and aimed to systematically evaluate the programme, identify areas for enhancement through feedback and coproduction with residents, and assess the impact on curriculum delivery.

**Methods:**

A mixed methods evaluation was undertaken for delivery of the theory paper teaching. Residents attending Paper A and Paper B MRCPsych teaching programmes in 2023–2024 (n=201) were asked to fill in post-session questionnaires comprising Likertscale items and some questions allowing free text answers. Quantitative data were pooled into descriptive categories, while qualitative responses underwent inductive content and thematic analysis. Themes were further explored and validated through resident focus groups and verified with a large language model for research triangulation. Insights informed a structured review of the 2024–2025 MRCPsych curriculum delivery involving a senior resident. The revised programme was reevaluated using the same methodology (n=237), enabling comparison.

**Results:**

In 2023–2024, residents rated content clarity, presentation quality and clinical relevance highly (≥83% “excellent” or “very good”). Suggestions included increased exam focus, more interactivity, improved time management, and provision of presession materials. Following curriculum revision, 2024–2025 ratings demonstrated further improvement, with ≥85% selecting “excellent” or “very good” across domains and reduction in “poor” responses. Candidates particularly valued enhanced exam-focused content, greater session interaction, and improved structuring and expansion of complex topics. At both time periods there was a request for increasing interaction in teaching and strengthening alignment with the MRCPsych syllabus.

**Conclusion:**

Co-produced evaluation enabled systematic identification of ‘areas of improvement’ within the MRCPsych teaching programme, allowing targeted changes that yielded measurable improvements in satisfaction. This approach demonstrates circular evaluation of curriculum delivery through a continuous feedback loop within a quality assurance model that integrates curriculum design, teaching delivery, assessment, and outcomes. We recommend continued collaborative, resident-centred refinement of MRCPsych courses with mapping to the syllabus.

